# GABA has a presynaptic inhibitory effect at *Lumbricus terrestris* body wall muscle synapses.

**DOI:** 10.17912/micropub.biology.001055

**Published:** 2023-11-30

**Authors:** William L. Coleman, Leah E. McCartney

**Affiliations:** 1 Biology, Commonwealth University of Pennsylvania- Bloomsburg. Bloomsburg, PA, USA

## Abstract

Earthworm body wall muscle synapses have been suggested to contain both excitatory and inhibitory inputs, and therefore allow for investigation of excitatory/inhibitory signaling in an easily accessible model system. While previous studies have focused on postsynaptic GABAergic inhibitory mechanisms, this study investigated the hypothesis that GABAergic signaling also has presynaptic inhibitory function. This hypothesis was tested by loading synaptogreen C4 dye (also called FM1-43) into presynaptic vesicles in the presence of GABA at *Lumbricus terrestris* longitudinal muscle synapses. GABA treatment significantly reduced the fluorescence intensity observed at these synapses, suggesting that GABAergic signaling does indeed have a presynaptic inhibitory mechanism.

**Figure 1. GABA treatment significantly reduced presynaptic synaptogreen C4 fluorescence intensity:**
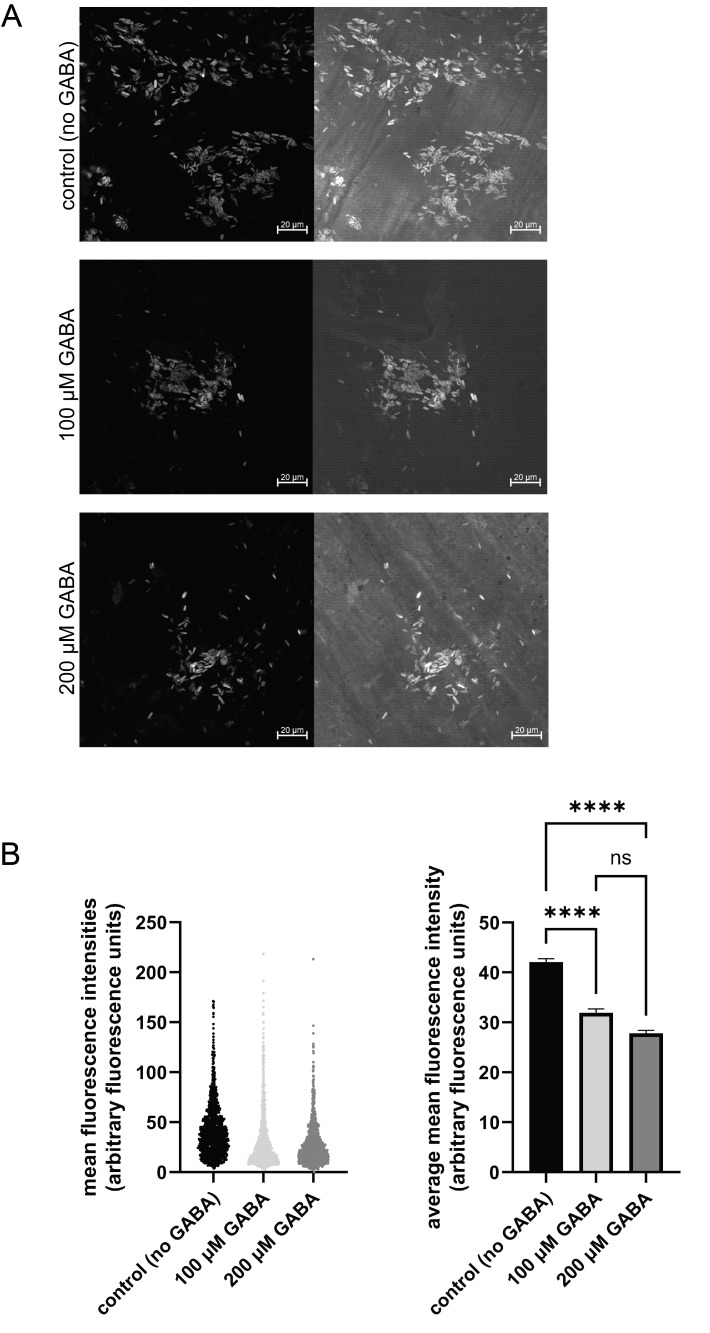
Panel A. Representative image sets of synaptogreen C4 staining from the different treatment groups: control (no GABA treatment), top; 100 µM GABA, middle; 200 µM GABA, bottom.&nbsp; Each set includes fluorescent staining only (left), and the fluorescent staining overlaid with the corresponding DIC imagefrom the same field (right). Scale bars are 20 µm.&nbsp; Panel B.&nbsp;Scatter plot of individual data points (left) and summary data (right) for the three treatment groups.&nbsp; Three earthworms were used for each treatment group, with data collected from seven to nine randomly selected confocal fields for each worm.&nbsp; The number of areas analyzed for each group were n = 1504 for control (no GABA treatment); n = 1391 for 100 µM GABA; and n = 1497 for 200 µM GABA.&nbsp; A Kruskal-Wallis test showed a statistically significant difference among the groups (p <0.0001).&nbsp; Dunn’s multiple comparison tests revealed that 100 µM GABA and 200 µM GABA both significantly reduced the fluorescence intensity of synaptogreen C4 staining compared to the control (no GABA) group (each with p < 0.0001), but that the two GABA treatment groups were not significantly different from each other (p > 0.05).

## Description

The balance of excitatory and inhibitory signaling is critical for normal nervous system function. Earthworm body wall muscle synapses have long been suggested to contain both excitatory and inhibitory inputs, and therefore allow for investigation of excitatory/inhibitory signaling in an easily accessible model system. Previous studies have largely focused on postsynaptic GABAergic inhibition at earthworm body wall muscle synapses. Investigations in the late 1960’s demonstrated that application of GABA caused hyperpolarization of the muscle, and that inhibitory potentials were blocked by the GABA _A_ inhibitor picrotoxin (Hidaka et al., 1969) . Another study also supported that GABAergic inhibition in earthworm muscles is mediated by chloride conductance (Ito et al., 1969) . More recent studies demonstrated that GABA hyperpolarizes resting membrane potentials in *Lumbricus terrestris* body wall muscles (Volkov et al., 2005; Volkov et al., 2006; Volkov et al., 2007; Volkov et al., 2011) , and that all of the components necessary for GABAergic signaling including GABA itself, the synthesizing enzyme GAD, GABA transporters, and subunits of both GABA _A_ and GABA _B_ receptors were present at these synapses (Nurullin et al., 2023) . In another common earthworm *Eisenia fetida* , GABA application had no effect on spontaneous muscle contractions, but caused relaxation of preparations that were precontracted by serotonin. Additionally, this result was replicated during treatment with the GABA _B_ agonist baclofen and was not blocked by the GABA _A_ antagonist bicuculline. The authors suggested that perhaps the inhibitory effect observed was by a presynaptic mechanism (Csoknya et al., 2005) . The purpose of this study was to investigate the hypothesis that GABA also has presynaptic inhibitory effects at the body wall muscle synapses in *Lumbricus terrestris* . To test this hypothesis, we loaded the styryl dye synaptogreen C4 (also called FM1-43) into presynaptic vesicles at longitudinal muscle synapses in the presence of exogenously applied GABA. The styryl dyes have been previously shown to stain presynaptic axon terminals (Betz and Bewick 1992) , and have since been used in many studies investigating presynaptic vesicle cycling and vesicle pool dynamics. The synaptogreen C4 staining in the earthworm longitudinal muscle appears as small, bright, oval fluorescent structures ( Fig. 1, panel A ), consistent with the staining pattern observed in other studies (Volkov et al., 2011; Volkov et al., 2013) . If GABA has a presynaptic inhibitory effect, it can be predicted that application of GABA during the loading of the dye would decrease the observed fluorescence intensity. Application of 100 µM GABA, a concentration shown to hyperpolarize the resting membrane potential (Volkov et al., 2011) , and 200 µM GABA both significantly decreased the synaptogreen C4 fluorescence intensity, although the two treatments were not significantly different from each other ( Fig. 1, panel B ). This result supports the hypothesis that GABA does indeed have a presynaptic inhibitory effect at the earthworm body wall muscle synapses. The observed decrease in fluorescence during GABA treatment is likely caused by an overall decrease in synaptic activity, leading to less vesicles taking up the synaptogreen dye. There is precedence for presynaptic GABAergic mechanisms including both ionotropic (GABA _A_ ) and metabotropic (GABA _B_ ) receptors in vertebrate organisms (Waldmeier and Baumann, 1990; Draguhn et al., 2008; Raiteri, 2008) , but also in crustaceans (Miwa et al., 1990; Parnas et al., 2000) , and in *Drosophila* (Yamagata et al., 2021; Raccuglia et al., 2016) . Future studies in earthworms can investigate the physiological role of this presynaptic mechanism and identify which specific GABA receptors mediate these presynaptic effects. The mechanism of the inhibitory effect can potentially be caused by chloride influx, such as from increased GABA _A_ receptor activity, or possibly the opening of potassium channels as a downstream effect of a GABA _B_ receptor mediated second messenger cascade. The contributions of the different GABAergic receptors to this presynaptic inhibitory mechanism can be investigated by loading synaptogreen in the presence of agonists (or antagonists) specific for the individual receptors and measuring changes in fluorescence intensity.

## Methods

Earthworms were purchased as *Lumbricus terrestris* from Carolina Biological Supply. Earthworms were pinned ventral side down to a dissecting tray. Posterior to the clitellum (typically several centimeters) worms were bisected longitudinally, the tissue spread and pinned, and digestive structures were removed. Throughout this process, the tissue was thoroughly rinsed with “normal” solution containing: 25 mM NaCl, 26 mM Na2SO4·10H2O, 6 mM CaCl2, 1 mM MgCl2·6H2O, 4 mM KCl, 2 mM Tris, 55 mM sucrose, and pH 7.3-7.4, similar to the recipe as found in (Drewes and Pax 1974) . 0.5 to 1 cm sections of this tissue were cut out and pinned to a sylgard lined dish. Under the dissecting microscope any remaining digestive tissues were removed, segmental nerve branches were clipped, and the ventral nerve cord was removed. The tissue was then re-pinned, and the areas adjacent to where the ventral nerve cord was normally located were cut away. In this way, most of the nephridia were also removed, and only the central section of tissue remained. Synaptogreen C4 dye (5 µM for all conditions, Biotium, Inc.) was loaded into presynaptic terminal areas using a depolarizing elevated potassium solution containing: 9 mM NaCl, 26 mM Na2SO4·10H2O, 6 mM CaCl2, 1 mM MgCl2·6H2O, 20 mM KCl, 2 mM Tris, 55 mM sucrose, and pH 7.3-7.4, for 10 minutes. This solution either contained no GABA (baseline/control staining), 100 µM GABA, or 200 µM GABA. Following three quick normal saline rinses to remove the elevated potassium solution, all tissue samples were treated with 100 µM SCAS (a chemical used to reduce background fluorescence, Biotium, Inc.) in normal saline for 8 minutes. After an additional quick rinse to remove the SCAS, tissue samples were taken for imaging on a ZEN LSM 800 confocal microscope (ZEISS). Samples were mounted in a drop of normal saline between two rectangular no. 2 cover slips (VWR). The tissue was secured and held flat by a round metal washer carefully placed on top of the cover slips. Images were acquired with a 40x water immersion objective lens, using a preset for FM1-43 in the ZEN software. Fluorescent areas were brought into focus and imaged as rapidly as possible without averaging. All images were taken using the same laser power and master voltage gain as the no GABA (baseline/control) condition. These values allowed for the capturing of a DIC field (using a TPMT channel) while not having excessive oversaturation in the fluorescence channel. Three separate worms were used for each of the three GABA conditions, and seven to nine randomly selected representative visual fields were captured from each worm. Negative control samples (not treated with synaptogreen C4) were utilized to assess any background fluorescence. These samples without synaptogreen C4 produced black images in the fluorescence channel. Images were analyzed using ZEN Blue software (ZEISS). Areas of synaptic staining were surrounded by rectangular selections and the mean fluorescence for the entire selection was recorded. Each rectangle typically contained multiple synaptic areas. Images shown were exported as tiff files directly from the ZEN Blue software, and were not adjusted for intensity in any way. Statistical analyses and graphs were made using GraphPad (Prism) software. As the data sets for each condition failed normality tests, non-parametric tests (Kruskal-Wallis followed by Dunn’s multiple comparisons) were utilized. The significance level for these tests was chosen as less than or equal to 0.05.

## Reagents

*Lumbricus terrestris* , Carolina Biological Supply Synaptogreen C4, Biotium, Inc. SCAS, Biotium, Inc.
